# The Effects and Mechanisms by which Saikosaponin-D Enhances the Sensitivity of Human Non-small Cell Lung Cancer Cells to Gefitinib

**DOI:** 10.7150/jca.30361

**Published:** 2019-10-22

**Authors:** Jian-cai Tang, Feng Long, Jia Zhao, Jia Hang, Yong-gang Ren, Jian-ye Chen, Bo Mu

**Affiliations:** 1 Department of Biochemistry, North of Si Chuan Medical College, Nan Chong, Si Chuan, China;; 2 Department of Pharmacy, Nan Chong Central Hospital, Nan Chong, Si Chuan, China;; 3 School of Pharmacy, North of Si Chuan Medical College, Nan Chong, Si Chuan, China.

**Keywords:** Saikosaponin-d, gefitinib resistance, STAT3, Bcl-2

## Abstract

Non-small cell lung cancer (NSCLC) patients with epidermal growth factor receptor (EGFR)-sensitive mutations benefit from epidermal growth factor receptor tyrosine kinase inhibitors (EGFR- TKIs). However, drug resistance is a major cause of therapeutic failure. This study examined whether saikosaponin-d (SSD) enhances the anti-tumor effect of gefitinib in NSCLC cells. Cell Counting Kit-8 (CCK-8) was used to determine cell viability. Cell apoptosis was examined by flow cytometry. Signal transducer and activator of transcription (STAT3), phosphor-STAT3 (P-STAT3), and B-cell lymphoma 2 (Bcl-2) were detected by Western blot. An HCC827/GR tumor model was established to observe the effect of combination therapy in vivo. The combination of SSD with gefitinib had an enhanced inhibitory effect by reducing cell viability and inducing cells apoptosis in NSCLC cells. Furthermore, SSD decreased and increased the expression of P-STAT3 and Bcl-2, respectively. Down-regulated STAT3 promoted the sensitivity of lung cancer cells to gefitinib. The results of animal experiments also showed that SSD enhanced the anti-tumor effect of gefitinib. These results indicated that the combination of SSD with gefitinib had an increased antitumor effect in NSCLC cells and that the molecular mechanisms were associated with the inhibition of STAT3/Bcl-2 signaling pathway. Our findings suggest a promising approach for the treatment of NSCLC patients with EGFR-TKI resistance.

## 1. Introduction

Lung cancer is a leading cause of cancer death among men and women worldwide^1^. Non-small-cell lung cancer (NSCLC) accounts for approximately 85% of lung cancer cases and represents a heterogeneous group of cancers consisting mainly of squamous cell carcinoma (SCC), adenocarcinoma (AC), and large-cell carcinoma^2^. The molecular mechanisms altered in NSCLC include the activation of oncogenes, such as K-ras and epidermal growth factor receptor (EGFR) 3. Mutations or over-expression of EGFR lead to a proliferative advantage. In recent years, drugs targeting EGFR have provided significant clinical benefits for the treatment of NSCLC. Gefitinib, a small molecular tyrosine kinase inhibitor (TKI), has a good therapeutic effect in EGFR-sensitive mutant NSCLC. However, the development of additional EGFR mutations, MET proto-oncogene receptor tyrosine kinase (MET) and hepatocyte growth factor (HGF) amplifications, insulin-like growth factor receptor (IGFR) and fibroblast growth factor receptor (FGFR) activation, etc. after prolonged use of these inhibitors may result in acquired drug resistance^4^,^5^.The molecular mechanisms of EGFR-TKI resistance are complex; thus, overcoming EGFR-TKI resistance remains a significant challenge.

Signal transducer and activator of transcription 3 (STAT3) plays a critical role in tumor development and mediates many cellular processes such as proliferation and apoptosis^6^. STAT3 activation is associated with receptor-associated kinases to form homo- or heterodimers that translocate to the cell nucleus and promote the expression of a variety of genes. STAT3 is an important candidate for cancer treatment. Recent reports also showed that STAT3 activation may play a critical role in EGFR-TKI resistance^7^, ^8^.

Saikosaponin-d 9 (SSD) is a major triterpenoid saponin derived from *Bupleurum falcatum L* with anti-inflammatory and anti-infectious effects^10^, ^11^. Several recent years reports have shown the strong anti-tumor activities of SSD in breast cancer, prostate cancer, hepatocellular carcinoma, etc.^12^, ^13^. The anti-tumor mechanisms of SSD may involve the induction of apoptosis and autophagic cell death. Furthermore, SSD has been shown to overcome chemo-resistance in several cancer cells by inhibiting NF-kappa B signaling^12^, ^14^. However, whether SSD can enhance the sensitivity of NSCLC cells to gefitinib and overcome EGFR-TKI resistance remains unknown.

The present study investigated whether the combination of SSD with gefitinib would have a synergic antitumor effect in NSCLC cells. HCC827 and HCC827/GR were used to examine the anti-tumor effect *in vitro* and *in vivo*. The results showed that combination therapy inhibited tumor cell proliferation and induced tumor cell apoptosis *in vitro*. An HCC827/GR tumor model was established to observe the antitumor effects *in vivo*. The results showed that the combination therapy decreased tumor burden and increased tumor cell apoptosis. To explore the underlying molecular mechanisms, we measured STAT3, phosphor-STAT3 (P-STAT3), and B-cell lymphoma 2 (Bcl-2) expression after administration. We found that the combination therapy reduced P- STAT3 expression and increased Bcl-2 expression. siRNA to STAT3 increased the sensitivity of NSCLC cells to gefitinib. These results indicated that SSD increased the antitumor effect of gefitinib in NSCLC cells by inhibiting the STAT3/Bcl-2 signaling pathway.

## 2. Materials and Methods

### 2.1. Cell lines and reagents

SSD (>98% by high-performance liquid chromatography [HPLC]) was provided by the Chengdu Institute of Biology of the Chinese Academy of Sciences. Gefitinib was obtained from Cayman (13166). Antibodies to STAT3, P-STAT3(Tyr705), and Bcl-2 were acquired from Abcam (USA). Antibodies to caspase-9, caspase-3, and cleaved caspase-3 were purchased from Cell Signaling Technology. Cell Counting Kit-8 (CCK-8) was obtained from Dojindo (Japan). Cell cycle apoptosis kits were purchased from Boster (China). The human NSCLC cell lines H1975 (EGFR-resistance mutation) and HCC827 (EGFR-sensitive) were provided by the American Type Culture Collection (ATCC). PC-9 (EGFR-sensitive) and HCC827/GR (gefitinib resistance) were obtained from Cell-Bio Company (Shanghai, China). HepG2 was obtained from the Cell Bank of the Shanghai Institutes for Biological Sciences, Chinese Academy of Sciences. Cells were cultured in 1640 media with 10% fetal bovine serum (FBS) at 37°C in a humidified atmosphere containing 5% CO_2._

### 2.2. CCK-8 assay

H1975, PC-9, HCC827, HCC827/GR, and HepG2 were seeded on 96-well plates and cultured overnight. The cells were treated with SSD or gefitinib or their combination. CCK-8 was used to detect cell viability according to the manufacturer's instructions.

### 2.3. Flow cytometry

HCC827 and HCC827/GR cells were seeded on six-well plates at 4×10^5^ cells per well. The cells were then treated with gefitinib, SSD, or their combination. Flow cytometry was used to determine cell apoptosis according to the manufacturer's instructions.

### 2.4. Western blot

The cells were treated with SSD or gefitinib or their combination for 24h. The total protein was extracted and separated by sodium dodecyl sulfate- polyacrylamide gel electrophoresis (SDS-PAGE), then transferred to a polyvinylidene difluoride (PVDF) membrane. The primary antibody was added and incubated at 4°C overnight. Next, a horseradish peroxidase (HRP)-conjugated secondary antibody was added and incubated for 1 hour. Protein blots were detected by enhanced chemiluminescence (ECL).

### 2.5. RNA interference

Targeting STAT3 siRNA and controls were purchased from Genepharma (Shanghai, China). Cells were transfected with siRNA using Lipofectamine 2000 Transfection Reagent according to the manufacturer's instructions. The sequences of siRNA targeting STAT3 and control were as follows:

- STAT3 siRNA sense: 5'-GGUACAUCAUGGGCUUUAUTT-3'
- Negative control sense: 5'-UUCUCC GAACGUGUCACGUTT-3'

### *2.6.* Animal experiments *in vivo*

An HCC827/GR tumor model was established to observe the anti-tumor effect *in vivo*. First, mice were intraperitoneally injected with HCC827/GR (1×10^7^) cells in the dorsal region (Vital River Laboratory Animal Technology, Co. Ltd, Beijing). When the tumor size reached 400-500 mm3, the mice were treated and the tumors were harvested. Single-cell suspensions were prepared from the tumor tissue by mechanical trituration. HCC827/GR (1×107) cells were intraperitoneally injected into the other nude mice. When tumor size reached 100 mm3, the mice were randomly divided into four groups of seven mice each, as follows: control (dimethyl sulfoxide [DMSO]), gefitinib, SSD (5 mg/kg/day) + gefitinib (50 mg/kg/day), SSD (10 mg/kg/day) + gefitinib (50 mg/kg/day). Treatment was performed for 14 days. Tumor volumes were evaluated every three days according to the following formula: V=0.52×width^2^×length.

### 2.7. Immunohistochemistry (IHC)

Tumor tissues were obtained from tumor-bearing nude mice 24h after the last treatment. The samples were first fixed with formalin (10%), and embedded in paraffin wax. Next, the tumor tissues were sectioned (4-5µm) and deparaffinized in xylene, rehydrated through reduced ethanol concentrations and washed with phosphate-buffered saline (PBS). Third, the sections were incubated overnight with P-STAT3 (1:100) and Bcl-2 (1:100) monoclonal antibodies at 4°C. Finally, an HRP-labeled second antibody was combined with the primary antibody and immunostaining was performed according to the manufacturer's instructions. The results were evaluated by counting the numbers of positive cells in six random fields.

### 2.8. Quantitative assessment of apoptosis

Tumor sections were prepared as described previously. The apoptosis of tumor tissues was assessed by terminal deoxynucleotidyl transferase-mediated deoxyuridine triphosphate-biotin nick-end labeling (TUNEL) using an in situ cell death detection kit (DeadEnd™ Fluorometric TUNEL System, Promega, USA). The number of TUNEL-positive cells was counted under a 200× magnification using a fluorescence microscope.

### 2.9. Statistical analysis

All data were analyzed using with SPSS Statistics for Windows, version 17.0. The results are presented as means±SD of three independent experiments. P<0.05 was considered to indicate statistically significant differences.

## 3. Results

### 3.1. SSD enhances the sensitivity of NSCLC cells to gefitinib

**Figure 1:**
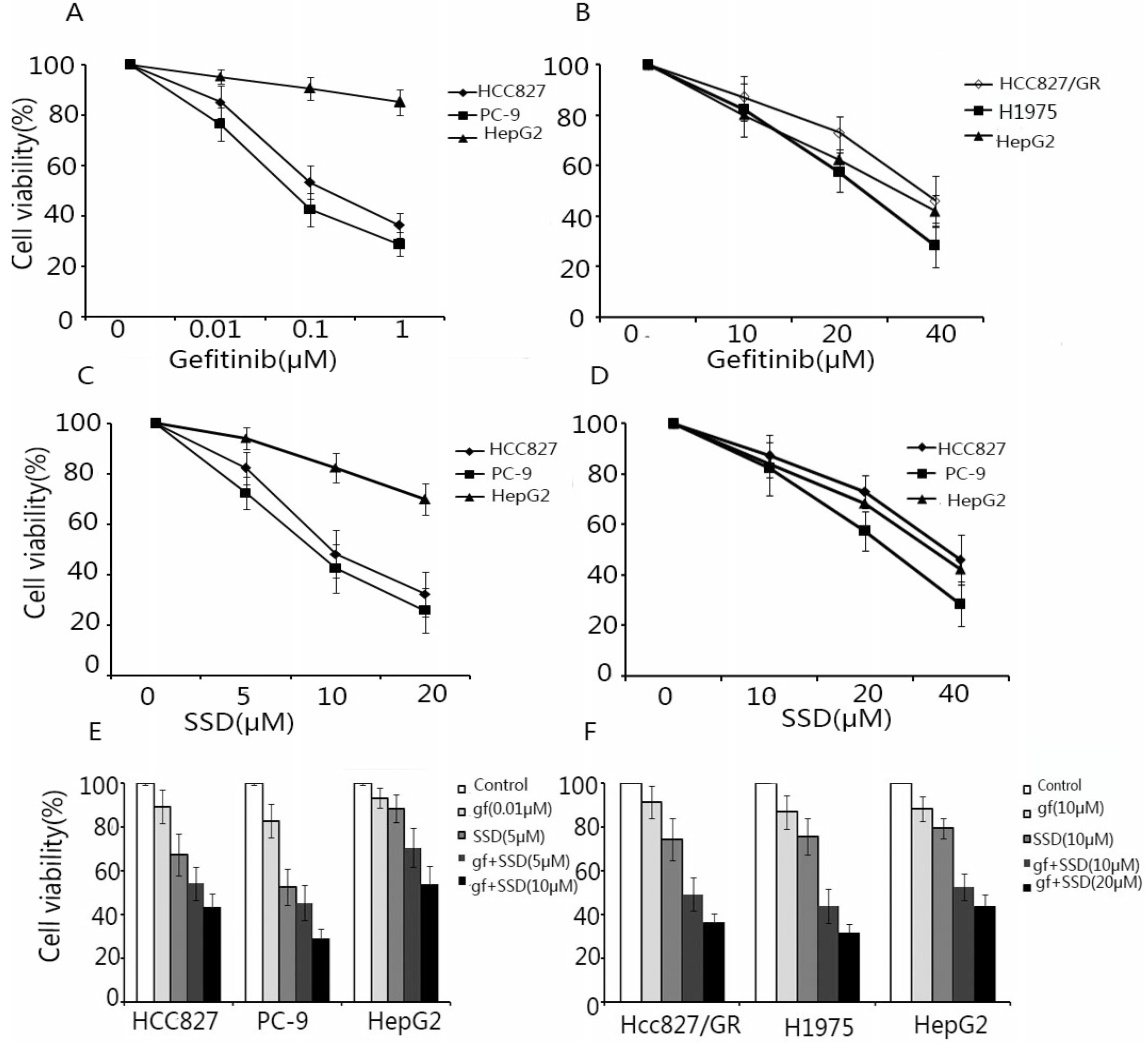
** Saikosaponin-d (SSD) augments the anti-tumor effect of gefitinib in EGFR-TKI-sensitive HCC827 and PC-9 and EGFR-TKI-resistant HCC827/GR and H1975 cells.** HCC827, PC-9, HCC827/GR, H1975, and HepG2 cells were seeded on 96-well plates at 5×10^3^ cells/well. Cells were treated with gefitinib (A,B) or SSD (C,D) alone or in combination (C,D) for 24 h. Cell viability was detected by Cell Counting Kit-8 (CCK-8). The results showed that SSD enhanced the sensitivity of non-small lung cancer cells to gefitinib in a dose-dependent manner.

To assess the effect of SSD on increasing the sensitivity of NSCLC cells to gefitinib, HCC827, PC-9, HCC827/GR, H1975, and HepG2 (external control) cells were treated with SSD or gefitinib or their combination. Cell viability was assayed by CCK-8. As shown in Fig. 1A and B HCC827 and PC-9 cells were sensitive to gefitinib, while HCC827/GR and H1975 cells showed resistance. As shown in Fig. 1C and D, SSD alone had an inhibitory effect of NSCLC cells. A low concentration of gefitinib combined with SSD showed that SSD increased the anti-tumor effect of gefitinib in NSCLC cells (Fig. 1E and F). These results demonstrated that SSD sensitized NSCLC cells to gefitinib.

### 3.2. SSD increases gefitinib-induced cell apoptosis

**Figure 2:**
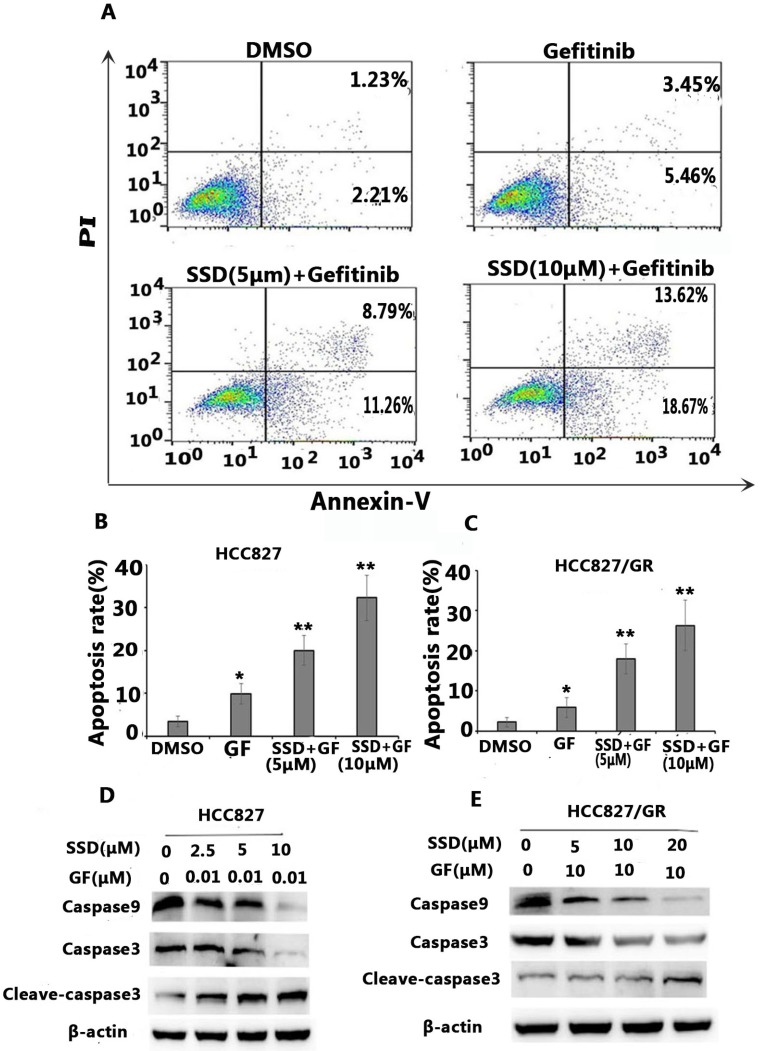
** SSD increases gefitinib-induced apoptosis.** HCC827 and HCC827/GR cells were treated with gefitinib or SSD their combination for 24 hours. Cell apoptosis was measured by flow cytometry after staining with Annexin-V/PI according to the manufacturer's instructions. A. Typical flow cytometric graph of apoptosis. B. The percentage of apoptosis after drug administration in HCC827 cells C. The percentage of apoptosis after drug administration in HCC827/GR cells. D,E. Caspase-3, cleaved caspase-3, and caspase-9 expression by Western blot.

To determine the possible mechanisms of the synergistic effect, cell apoptosis was assessed by flow cytometry. Apoptosis-related proteins were detected by Western blot. As shown in Figure 2A-C, SSD significantly augmented gefitinib-induced apoptosis in HCC827 and HCC827/GR cells. Figure 2D shows decreased levels of caspase-9 and caspase-3 in the SSD combined with gefitinib groups, while cleaved caspase-3 was significantly increased. These results indicated that SSD induced tumor cell apoptosis by enhancing caspase-3 activity.

### 3.3. SSD inhibits gefitinib-induced STAT3 activation

**Figure 3:**
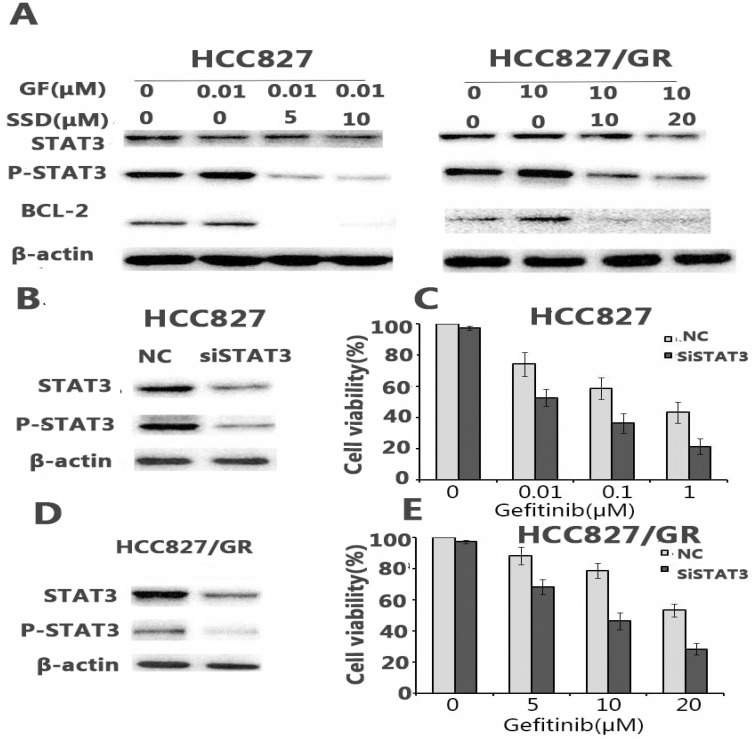
** SSD inhibits gefitinib-induced STAT3 activation.** A. STAT3, p-STAT3, and Bcl-2 expression after drug administration. P-STAT3 expression increased after gefitinib treatment, while combination therapy decreased expression of P-STAT3 and Bcl-2. B,D. siSTAT3 treatment in HCC827 and HCC827/GR cells. siSTAT3 enhanced the sensitivity of non-small cell lung cancer cells to gefitinib (*p <0.05,**p <0.01). C,E. Cell viability was assessed by Cell Counting Kit-8 (CCK-8) after siRNA STAT3 treatment.

Aberrant STAT3 activation may play an important role in the development of drug resistance. We found that gefitinib could feedback-activate STAT3 in HCC827 and HCC827/GR cells. As shown in Figure 3A, P-STAT3 was increased after gefitinib treatment. SSD decreased P-STAT3 and downstream Bcl- 2 expression (Fig. 3A).

To further determine the key role of STAT3 in SSD overcoming gefitinib resistance, sensitive HCC827 cells and HCC827/GR cells with acquired resistance were transfected with siRNA against STAT3. The results showed that down-regulating STAT3 led to a significant increase in gefitinib-induced growth inhibition (Fig. 3C, E).

### 3.4. SSD enhances the antitumor effect of gefitinib *in vivo*

**Figure 4:**
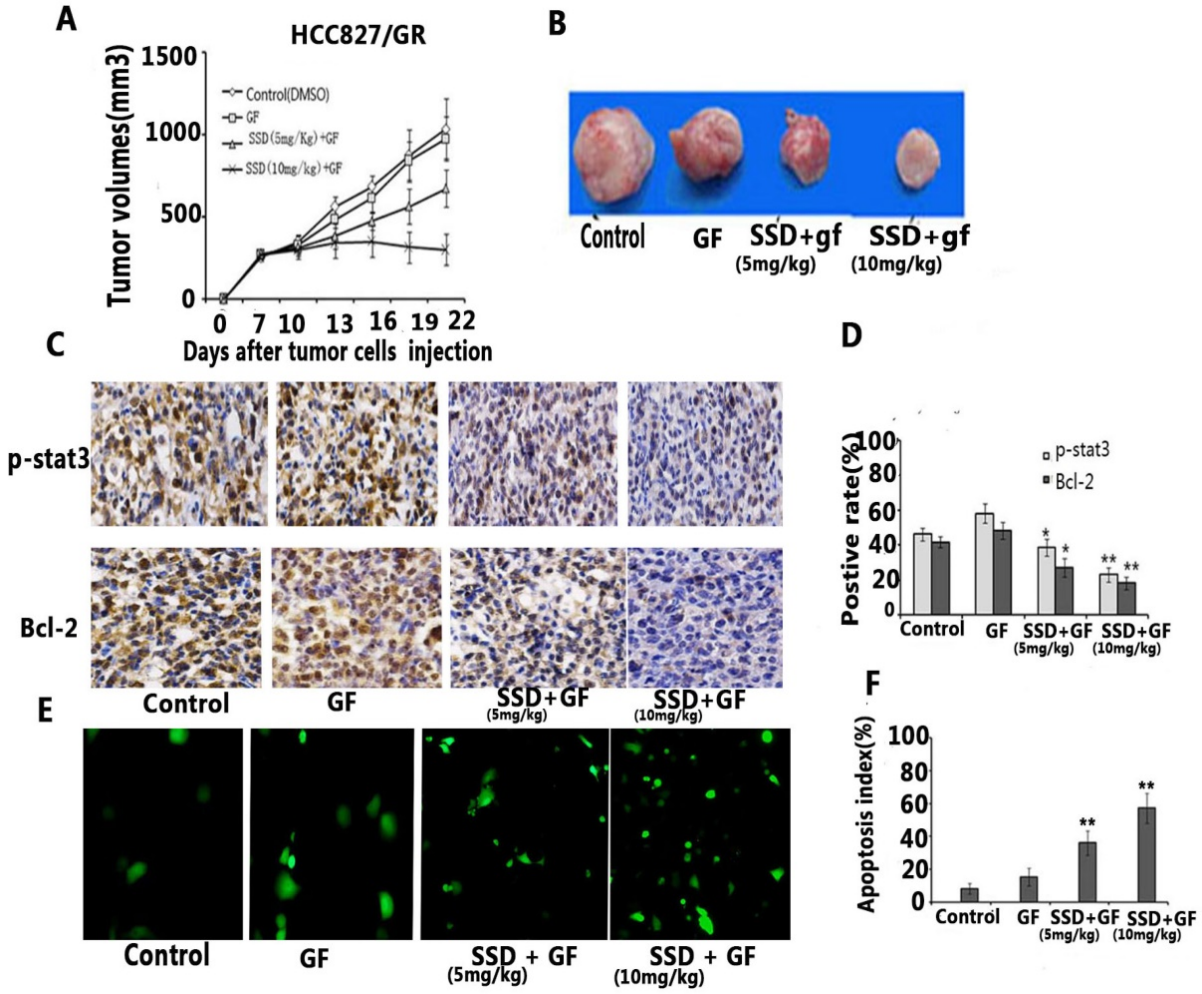
** SSD enhances the anti-tumor effect of gefitinib in *vivo*.** An HCC827/GR tumor model was developed to observe the anti-tumor effect of SSD in combination with gefitinib *in vivo.* Mice were injected with 1×10^7^ HCC827/GR cells. Seven days after tumor cell injection, the mice were randomly divided into four groups: control (DMSO), gefitinib (50 mg/kg/day), SSD( 5mg/kg/day) + gefitinib (50 mg/kg/day), and SSD (10 mg/kg/day) + gefitinib (50 mg/kg/day). The treatment was performed for 14 days at the same time (n=7 per group) A. The combination therapy inhibited tumor growth compared to the control or gefitinib-only treatment groups (n=7, p <0.01). Data are shown as means ± SD.B. Representative tumor image. C. Typical image of immunohistochemistry (IHC) staining of P-STAT3 and Bcl-2 in tumor tissues (×200). D. Average staining intensities of p-STAT3 and Bcl-2 evaluated according to the number of positive cells in six random fields. The results showed significantly decreased expression of p-STAT3 and Bcl-2 in the combination therapy group (**P<0.01) E. Representative image of TUNEL assay in tumor tissue. F. Apoptotic index within tissues. The combination therapy displayed a significant increase in the number of apoptotic cells in the tumor tissues compared to those in control or single gefitinib groups.

To further assess the synergistic effect on promoting NSCLC cell apoptosis *in vivo*, and HCC827/GR tumor model was established in xenograft mice. We found that gefitinib had no anti-cancer activity on acquired resistance in HCC827/GR, although the dose of gefitinib reached 50 mg/kg/day for two weeks. Combined gefitinib with SSD showed an inhibitory effect *in vivo* (Fig 4A,B). Furthermore, gefitinib treatment resulted in increased P-STAT3 and Bcl-2 expression, while SSD decreased the levels of P-STAT3 and Bcl-2 (Fig. 4C and D). Finally, tumor cell apoptosis was detected by TUNEL assay. As shown in Fig. 4E and F, the combination therapy increased the rate of tumor cell apoptosis. These results indicated that SSD may overcome gefitinib resistance by inhibition of the STAT3/Bcl-2 signaling pathway.

An HCC827/GR tumor model was developed to observe the anti-tumor effect of SSD in combination with gefitinib *in vivo.* Mice were injected with 1×10^7^ HCC827/GR cells. Seven days after tumor cell injection, the mice were randomly divided into four groups: control (DMSO), gefitinib (50 mg/kg/day), SSD( 5mg/kg/day) + gefitinib (50 mg/kg/day), and SSD (10 mg/kg/day) + gefitinib (50 mg/kg/day). The treatment was performed for 14 days at the same time (n=7 per group) A. The combination therapy inhibited tumor growth compared to the control or gefitinib-only treatment groups (n=7, p <0.01). Data are shown as means ± SD.B. Representative tumor image. C. Typical image of immunohistochemistry (IHC) staining of P-STAT3 and Bcl-2 in tumor tissues (×200). D. Average staining intensities of p-STAT3 and Bcl-2 evaluated according to the number of positive cells in six random fields. The results showed significantly decreased expression of p-STAT3 and Bcl-2 in the combination therapy group (**P<0.01) E. Representative image of TUNEL assay in tumor tissue. F. Apoptotic index within tissues. The combination therapy displayed a significant increase in the number of apoptotic cells in the tumor tissues compared to those in control or single gefitinib groups.

## 4. Discussion

NSCLC patients with EGFR-sensitive mutations have excellent responses to EGFR-TKIs^15^. However, most patients eventually develop resistance to the TKIs, resulting in treatment failure^16^. Thus, there is an urgent need to develop a new strategy. In the present study, SSD enhanced the sensitivity of NSCLC cells to gefitinib in *vitro* and in *vivo*. The underlying molecular mechanisms may be associated with inhibition of the STAT3/Bcl-2 signal pathway. siRNA to STAT3 increased the anti-tumor effect of gefitinib in NSCLC cells. These data provide a new approach for overcoming EGFR-TKI resistance.

SSD, isolated from *Bupleurum falcatum L,* has been widely used for its anti-inflammatory and anti-infectious disease effects 17 Recent reports have demonstrated the anti-tumor activity of SSD in several types of cancer^18^-^20^. One report showed that SSD sensitizes chemoresistant ovarian cancer cells to cisplatin-induced apoptosis by facilitating mitochondrial fission and G2/M arrest^21^. Another report concluded that SSD enhances the anticancer potency of TNF-alpha by overcoming its undesirable response of activating NF-kappa B signaling in cancer cells^12^. The results of these studies implied that SSD may be effective for overcoming multidrug resistance. In the present study, SSD enhanced the anti-tumor effect of gefitinib in NSCLC both*.in vitro* and *in vivo*. To our knowledge, ours is the first study to show that SSD enhances the sensitivity of NSCLC cells to gefitinib.

Regarding the possible molecular mechanisms, we found that enhanced STAT3 activation resulted in increased Bcl-2 expression after gefitinib treatment (Fig 3A), which may be an important factor in primary or acquired resistance in NSCLC cells. Some reports have shown that STAT3 activation promotes tumor cell survival under stress condition and is an attractive target in multiple cancers^22^-^24^. Our results are consistent with those previously reported^25^. However, the total levels of STAT3 did not change after drug treatment. Therefore, the drug may not affect STAT3 expression but may instead play an important role in STAT3 phosphorylation. Furthermore, we also observed that the combination of SSD with gefitinib decreased P-STAT3 and Bcl-2 expression, indicating that SSD can inhibit gefitinib-induced P-STAT3/Bcl-2 signal pathway activation. SiStat3 enhanced gefitinib-induced tumor cell apoptosis, as shown in Figure 3. *In vivo*, we also found that combination therapy decreased tumor burden and promoted tumor cell apoptosis. P-STAT3 and Bcl-2 were reduced after combination therapy (Fig 4). These data suggest that the potential molecular mechanism of SSD sensitization may be associated with an inhibition of STAT3 activation.

In conclusion, our results showed that Saikosaponin-d can boost the antitumor effect of gefitinib in NSCLC cells both *in vitro* and *in vivo*. The underlying molecular mechanisms may be associated with the induction of tumor cell apoptosis by inhibition of the STAT3/Bcl-2 signal pathway. Furthermore, we observed that combination therapy had no evident adverse effect *in vivo*. These results indicated that the combination Saikosaponin-d with gefitinib may be a promising approach for NSCLS treatment However; we cannot fully confirm the direct molecular mechanism of Saikosaponin-d resulting in decreased P-STAT3 and Bcl-2. Future studies are required to elucidate these mechanisms. The results of the present study demonstrate a novel strategy for overcoming gefitinib resistance.
